# Mathematical modeling of an oscillating gene circuit to unravel the circadian clock network of *Arabidopsis thaliana*

**DOI:** 10.3389/fpls.2013.00003

**Published:** 2013-01-25

**Authors:** Nora Bujdoso, Seth J. Davis

**Affiliations:** Department of Plant Developmental Biology, Max Planck Institute for Plant Breeding ResearchCologne, Germany

**Keywords:** *Arabidopsis thaliana*, circadian clock, mathematical modeling, light signal transduction, temperature acclimation, hormone signal integration, metabolic signal integration, stress signal integration

## Abstract

The *Arabidopsis thaliana* circadian clock is an interconnected network highly tractable to systems approaches. Most elements in the transcriptional–translational oscillator were identified by genetic means and the expression of clock genes in various mutants led to the founding hypothesis of a positive–negative feedback loop being the core clock. The identification of additional clock genes beyond those defined in the core led to the use of systems approaches to decipher this angiosperm oscillator circuit. Kinetic modeling was first used to explain periodicity effects of various circadian mutants. This conformed in a flexible way to experimental details. Such observations allowed a recursive use of hypothesis generating from modeling, followed by experimental corroboration. More recently, the biochemical finding of new description of a DNA-binding activity for one class of clock components directed improvements in feature generation, one of which revealed that the core of the oscillator is a negative–negative feedback loop. The recursive use of modeling and experimental validation has thus revealed many essential transcriptional components that drive negative arms in the circadian oscillator. What awaits is to more fully describe the positive arms and an understanding of how additional pathways converge on the clock.

## A HISTORICAL PATH THROUGH THE MOLECULAR GENETICS OF THE PLANT CIRCADIAN CLOCK

The circadian clocks of various phyla have a rich history of modeling in molecular terms. The discovery of the first *Drosophila* clock gene *PERIOD* led to early mechanistic models of a daily oscillator (Goldbeter, 1995). As more genes were added to this animal oscillator, models became more rich and predictive (Gonze et al., 2011). Similar modeling approaches have been applied to the fungal (Tseng et al., 2012), the mammalian (Gerard and Goldbeter, 2012), and the cyanobacterial oscillators (Hatakeyama and Kaneko, 2012). Molecular differences in circadian clocks have been visible, which suggests multiple evolutionary origins (Dunlap et al., 2004).

**FIGURE 1:**
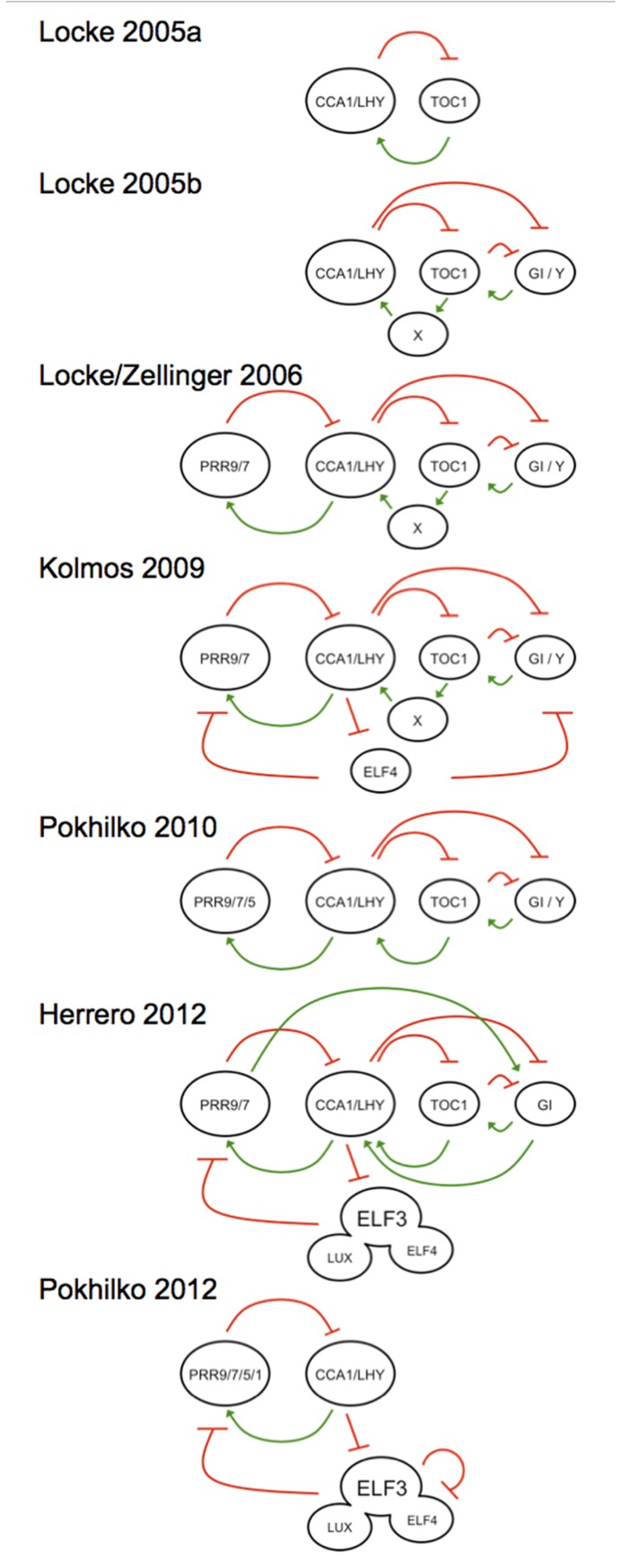
**Graphical outline of the mathematical *A. thaliana* clock models in historical order, showing the development from a simple positive–negative feedback model (Locke et al., 2005a) toward more complicated interconnected feedback loops (Locke et al., 2005b, 2006; Zeilinger et al., 2006; Pokhilko et al., 2010)**. The oscillator expands with he subsequent placement of an evening complex element (Kolmos et al., 2009). Recent experiments could prove the existence of an evening complex working directly on the morning loop (Herrero et al., 2012; Pokhilko et al., 2012). Activation elements are defined by a green positive arrow and a red negative arrow indicates repression, respectively. All models are centered at the CCA1/LHY hub to illustrate the directional movement of the models over publication time. Each model is described in the text and the models are named after the primary author and publication year. Note that two models define the 2006 efforts, and are the predominant models that has driven most recent circadian work.

For plants, a similarly rich use of modeling can be found (Shin and Davis, 2010). To date no less than seven distinct models of the plant clock have been published (**Figure 1**), and the most recent kinetic model (Pokhilko et al., 2012) and linear time invariant (LTI) model (Herrero et al., 2012) have highlighted the complexity in this system. These models, alongside other approaches, such as Boolean modeling (Akman et al., 2012), have various strengths in predicting the system. The first model that placed the plant clock in molecular terms was in 2005 (Locke et al., 2005a), where the authors presented an approach to inferring models when available molecular-genetic data are sparse and noisy. It is of interest that the recursive use of molecular genetics with mathematical modeling has served as a platform toward rational genetic understanding of the gears that make up the plant clock. Such iterative approaches should also bridge the transition of knowledge from genetic to cellular terms. In this review, we will briefly overview the important use of mathematics in unraveling the plant circadian oscillator, in an integrative sense, sometimes with “false starts,” to define where the state of the art is now. The need for new models will also be defended.

## THE IMPORTANCE OF DAILY RHYTHMS IN PLANTS

The circadian clock drives rhythms of growth and development as a pervasive force in essentially all aspects of a plant life cycle (Davis and Millar, 2001). For example, primary and secondary metabolism is timed to be coincident with available photic-energy sources and predictable heat and cold (McClung and Davis, 2010; Sanchez et al., 2011). These environmental rhythms are all a consequence of the earth’s rotation, and a plant has a fitness benefit to anticipate all three (Green et al., 2002; Dodd et al., 2005; Yerushalmi et al., 2011).

The assembly of a coherent understanding of the plant clock required suitable assay systems to, with limited intervention, measure various parameters of clock function. Oscillations can be mathematically characterized by period, phase, and amplitude. Additionally, the precision of the circadian oscillation has been described by its faithfulness to maintain constant periodicity (all parameters are graphically described in the supplement of Hanano et al., 2006). To characterize circadian mutants, two robust systems were initially employed in the model plant *Arabidopsis thaliana*: luciferase reporter rhythms and leaf movement rhythms (Millar et al., 1995; Swarup et al., 1999). By analyzing circadian parameters of mutants, genetic approaches have uncovered a number of genes in the clock, and these will be described below.

## THE *Arabidopsis thaliana* CLOCK MODELS

The first mutants defective in clock function provided a platform toward understanding the components of this oscillating gene network (Millar et al., 1995). One break-through in this approach led to the hypothesis that the morning acting clock genes *CIRCADIAN CLOCK ASSOCIATED 1* (*CCA1*) and *LATE ELONGATED HYPOCOTYL* (*LHY; *Schaffer et al., 1998; Wang and Tobin, 1998) repress the evening acting transcriptional regulator *TIME OF CAB EXPRESSION (TOC1*; Strayer et al., 2000), which was then reported to induce *CCA1* and *LHY* expression (Alabadi et al., 2001). This defined a so-called negative–positive feedback loop as the core oscillator (graphically illustrated in the Locke et al., 2005a model; **Figure 1**). Early genetic models of the higher plant clock realized the lack of construction features of this oscillator (McWatters et al., 2001; Staiger, 2002). Mathematical modeling of this network revealed that the clock network must be more complicated (Locke et al., 2005a). Nevertheless, this “one-loop” model provided a critical conceptual framework that guided a decade of molecular-genetics research. From this core-loop hypothesis, many other clock genes were described and placed to the clock circuitry.

Experimental data on the clock accumulated rapidly and exceeded the conceptual capacity to “understand” the network. Several landmark papers in systems biology resolved this dilemma. Firstly it was mathematically hypothesized that the oscillator is composed of interconnected loops (Locke et al., 2005b model; **Figure 1**). Then work from the Doyle and Millar groups separately defined similar kinetic models that incorporated most available molecular-genetic data (Locke et al., 2006; Zeilinger et al., 2006). These models took in to account the period lengthening and shortening behavior of mutations in genes defined in these models, and were often capable of recapitulating the transcript misexpression levels of genes in the clock in various reciprocal mutant combinations. Both models came to similar conclusions of how the multiple interconnected feedback loops are constructed (Locke/Zellinger 2006 model; **Figure 1**; Locke et al., 2006; Zeilinger et al., 2006). These working hypotheses were predictive for future experiments, and subsequent molecular-genetic tests have often conformed to mathematical predictions. One key observation predicted was that the *cca1 lhy toc1* triple mutant, which lacks the core oscillator, would be arrhythmic. That was indeed experimentally observed (Ding et al., 2007; Ito et al., 2007b). Taken together, the seminal hypothesis of Doyle and Millar that the oscillator is a set of interconnected feedback loops defined for the first time a rational view of how plants tell daily time (Locke et al., 2006; Zeilinger et al., 2006). These explicit mathematical hypotheses have largely stood experimental tests (Shin and Davis, 2010), with some small additions and modifications described below, and one very large one (see below on TOC1 as a repressor).

The clock has been proposed as an interconnected feedback loop with morning, mid-day, evening, and night elements. The first practical models of the clock are illustrated in the Locke/Zellinger 2006 model (**Figure 1**; Locke et al., 2006; Zeilinger et al., 2006). In this, the morning expressed PSEUDORESPONSE-REGULATOR 9 and 7 (PRR9, PRR7) proteins repress *CCA1* and *LHY* expression, whose proteins in turn activate the former. This is the so-called morning arm. TOC1 protein in turn represses its activator “Y,” an element whose activity is at the end of the day, and this can be partially ascribed with the GIGANTEA protein (GI). This has been defined as the evening arm. These models served the clock community well, although some conflicts could be noted. For instance, the Locke/Zellinger 2006 model predicted that *Y*/*GI* transcript levels would elevate in the *toc1* null, but this could not be experimentally observed (Martin-Tryon et al., 2007). One explanation for this was that decreased *GI* expression in *toc1* loss-of-function mutants is not direct (Martin-Tryon et al., 2007), and this hypothesis still awaits testing. Finally, several groups have concluded that *GI* has biochemically separable roles in its ability to integrate light signals, work in the clock, and control flowering time (Mizoguchi et al., 2005; Martin-Tryon et al., 2007; Oliverio et al., 2007). How *GI* transcriptionally fits into the clock has not been particularly well resolved, but it has been proposed to additionally work in the clock as a hub of a protein destruction complex (Kim et al., 2007).

The Locke/Zellinger 2006 models considered multiple interconnections in the oscillating circuit. One reason for this was based on the observation that none of the founding clock components in this model were arrhythmic when mutated to loss-of-function. Genetic ablation of any one loop leads to the persistence of other loops; rhythms thus persist. In an interconnected circuit, reduction of paths reduces flux. As such, *cca1*, *lhy*, and* toc1* mutants were short period because there were less paths in the circuit (Locke et al., 2005a, 2006; Zeilinger et al., 2006). This could be extended in the Pokhilko et al. (2010) model (**Figure 1**). These described models thus did not allow for loss of function in any one gene to lead to arrhythmicity.

The notion of a single gene in the clock leading exclusively to periodicity defects needed to be reevaluated with the finding that the *EARLY FLOWERING 4* (ELF4) gene (Doyle et al., 2002) was core to the oscillator, and that when mutated, the oscillator stopped (Kolmos and Davis, 2007; McWatters et al., 2007). The *ELF4 *gene was found to be both necessary and sufficient to promote *CCA1* and *LHY*, and repress *TOC1* (McWatters et al., 2007). This led to a preliminary hypothesis that ELF4 worked directly on these genes (Kolmos and Davis, 2007). That hypothesis could quickly be refuted. If *CCA1*/*LHY* levels were low and *TOC1* levels were high in *elf4*, then it was a simple expectation that *PRR9*, *PRR7*, and *GI* levels would also be low. Experimentally the reverse was found for all cases (Kolmos et al., 2009). The Locke et al., 2006 model helped to solve this contradiction. Using parameter fitting of the observed levels of *PRR9*, *PRR7*, and *GI* in *elf4*, it was found that the oscillator would stop and that *CCA1* levels would collapse and that *TOC1* levels would be constantly high without rhythm. This is indeed exactly what is seen in the *elf4* mutant (Kolmos et al., 2009). Partial function alleles at *ELF4* conformed to this finding (Kolmos et al., 2009). Thus, *in silico* hypothesis testing of the Locke et al., 2006 model provided the first correct placement of an evening complex (EC) component into the oscillator (Kolmos et al., 2009 model). What this work did not address was the placement of *ELF4* in mathematical terms to this oscillating circuit.

## PLACING THE *ELF3* AND *LUX* EVENING COMPONENTS INTO THE CLOCK MODEL

The multiple interconnected feedback loops were made in part to accommodate the phenotypic effects from loss-of-function data. Arrhythmic mutants could not simply be defined in the original Doyle and Millar models (Locke et al., 2006; Zeilinger et al., 2006). Arrhythmic mutants exist at three loci and these are at the EC-components *ELF4*, *ELF3*, and *LUX* (Hazen et al., 2005; McWatters et al., 2007; Thines and Harmon, 2010). None of these genes had been conceptualized in the core-oscillator mechanism. ELF4 association to ELF3 directs LUX action in the clock (Herrero and Davis, 2012; Herrero et al., 2012), and this complex was termed the EC (Nusinow et al., 2011).

Using LTI modeling, ELF4 and ELF3 were concluded to directly target *PRR9* and *PRR7* (Herrero et al., 2012 model; **Figure 1**). Indeed, the *elf3* mutant was found to be responsive to loss-of-function as it showed increased levels of these transcripts, especially in darkness, but not to such an extent under light (Kolmos et al., 2011). The *elf4* mutant had a larger effect on transcript misexpression phenotypes than *elf3*, and this was especially seen for the increase of *PRR7* transcript levels in *elf4* during the light phase, whereas in darkness, both *PRR9* and *PRR7* were similarly increased in *elf4* (Kolmos et al., 2009). Also, epistasis experiments showed that both *ELF3* and *LUX* act downstream of *ELF4* (Herrero et al., 2012). Consistent with that, whereas the ELF4 and ELF3 proteins have both been shown capable associated to the *PRR9* promoter (Dixon et al., 2011; Herrero et al., 2012), as can LUX (Helfer et al., 2011), only ELF4 has been shown to directly bind to the *PRR7* promoter (Dixon et al., 2011). Notably ELF4 over-expression resulted in attenuated *PRR7* accumulation to a reduced extent than that of ELF3 over-expression (Herrero et al., 2012). ELF4 thus appears to have more targets in the clock than ELF3 and LUX.

A systems analysis of the EC led to a new kinetic model that agreed with the LTI modeling of ELF4 and ELF3. Here a “repressilator” hypothesis was created with sequential waves of repression first by the transcription factors CCA1 and LHY, then by the PRRs, and finally by the EC, with LUX as the as the DNA-binding component of this complex (Pokhilko et al., 2012 model; **Figure 1**). Notable here was the biochemical finding that all PRRs directly associate to DNA (Gendron et al., 2012) and direct repression at their targets (Gendron et al., 2012; Huang et al., 2012). It is thought that all three EC genes are evening expressed because of direct repression by CCA1 and LHY (Kolmos and Davis, 2007; Lu et al., 2012; Pokhilko et al., 2012). The EC in turn is known to repress *PRR9* directly (Dixon et al., 2011; Helfer et al., 2011; Herrero et al., 2012), and genetically, can repress *PRR7* and perhaps *GI* (Kolmos et al., 2009, 2011; Herrero et al., 2012). It is currently unclear if the EC can directly repress *GI*, as mathematically predicted in one study (Kolmos et al., 2009), or if this is indirect through effects at PRR9 and PRR7, as mathematically predicted in another (Herrero et al., 2012). In the one test of this latter mathematical hypothesis, the *prr9 prr7* mutant was not found to have altered mean transcript levels of *GI* (Salome et al., 2010). Therefore, how *GI* fits in the clock is not particularly well understood within a transcriptional context.

## CONCEPTUAL USES OF MODELS: WEATHER PATTERNS AS AN EXAMPLE

Having a firm and experimentally validated model at hand allows for future optimization to test the robustness of the complex circadian clock network hypotheses. Here, the Millar group asked which environmental cues and following downstream regulations demanded such a highly complex clock network and followed an *in silico* approach to test whether the proposed oscillator is plausible in contexts of environmental variation seen in nature. For instance, they selected for networks that correctly predicted particular phases of the day under a light/dark cycle (Troein et al., 2009). The general conclusion was that changes in environmental cues demand for a high complexity in the clock network in order to encompass the details of environmental perturbations typical of daily weather or annual photoperiod variation. This finding provided a validation for the benefits of modeling. Tests of mathematical models often show their limitations. Community willingness to flexibly perform molecular-genetic and biochemical tests of these models has allowed for new model generation to account for such proven discrepancies.

While the existing models reveal a rational view of how an oscillating circuit can resist weather-related environmental changes, and still be sensitive to daily entrainment cues, the models have also been insightful into placing the vernalization effect on clock speed. Prolonged cold of winter results in the derepression of MADS domain transcription factors, of which the most notable is *FLOWERING LOCUS C* (*FLC*; Michaels and Amasino, 1999). Its expression correlates with period length, as *flc* is described as a short-period mutant and *FLC* over-expression displays a long period (Salathia et al., 2006). As predicted, vernalization effects that lower *FLC* transcript accumulation lead to a decrease in period length. This is genetically partially dependent on the allelic state of *FLC* (Salathia et al., 2006). From this, modeling could predict that this FLC action on the clock is through the expression of the EC component *LUX* (Edwards et al., 2006). Whether FLC binds to the *LUX* promoter, and whether FLC regulates the other EC components, has not yet been established.

## AN EXAMPLE OF A TECHNICAL LIMITATION OF MODELS: TOC1 AS A REPRESSOR

It was shown that TOC1 loss-of-function results in low *CCA1* and *LHY* transcript levels, which implies TOC1 as a transcriptional activator. However, TOC1 over-expression also results in low *CCA1/LHY* expression (Makino et al., 2002; Mas et al., 2003). Recent publications resolved this discrepancy and have shown that TOC1 acts as a transcriptional repressor of *CCA1* and *LHY*, as well as of *PRR9*, *PRR7*, and *GI* (Gendron et al., 2012; Huang et al., 2012; Pokhilko et al., 2012). Pokhilko et al. (2012) recently modeled this effect in kinetic terms, and included the EC elements, using the data of the *cca1/lhy* double mutant, in which only the evening loop sustains rhythmicity. They also included in this the post-translational modification of ELF3 by the ubiquitin E3 ligase COP1 and modulation of the EC complex by GI and ZEITLUPE (ZTL) (Kevei et al., 2006; Kim et al., 2007; Yu et al., 2008). This Pokhilko et al. (2012) model revealed consistency with the observed *ztl* and *prr9*/7 double mutant data, when TOC1 was included as an inhibitor for CCA1/LHY, which resolved the inconsistency of the previously proposed activator role of TOC1 and the available experimental data (Pokhilko et al., 2012).

Conclusively, the initial model of the oscillator with reciprocal transcriptional positive–negative feedback loops has been revised. Indeed all PRR-proteins have now been shown biochemically and molecularly to possess repressive function (Nakamichi et al., 2010; Gendron et al., 2012; Huang et al., 2012). This allows for current modeling efforts to define numerous negative–negative feedbacks. In the current Pokhilko et al. (2012) kinetic model, only CCA1 and LHY are defined as positive elements. This model also predicts that the EC controls *CCA1*/*LHY* and *TOC1* expression through the multi-loop of PRRs, which is consistent with the previous experimental observation of higher transcript levels of the *CCA1*, *LHY*, *TOC1*, and *PRR9* in the *elf3*, *elf4*, and *lux* mutants. Consequently, the Pokhilko et al. (2012) model shows the importance of repression of *TOC1* and *PRR9* by the EC for robust anticipation of dawn.

The recent work of Pokhilko et al. (2012) additionally considered the role of light as an input factor to the clock when investigating the phase change of the clock by light pulses. This model predicted that the acute activation of *CCA1*/*LHY* expression by light is required for the observed phase advance or delays at a given time during the night. The ability of the model to predict such an observation emphasizes the importance of incorporation of input signals to circadian modeling. From this, we need to start investigating effects of feedback signals from further downstream processes, such as hormonal signaling (Hanano et al., 2006) and as a consequence of metabolic changes (Dalchau et al., 2011), and cellular coordination of said processes (Wenden et al., 2012).

A general conclusion of recent kinetic and linear models could lead one to consider the clock network as “solved.” This is not the case. Two-component limit cycle oscillators can exist if at least one component is “autocatalytic” and there is also a negative feedback (Tyson, 2002; Novak and Tyson, 2008). Here, “limit cycle” means that every cycle is the same, and thus, there is no dampening or noise. If the plant circadian oscillator is not built in such a way, to make this oscillator, the circuit is anticipated to have a minimum of three components and positive and negative arms must exist within it: repressor networks need activators (Sprinzak and Elowitz, 2005). It is thus plausible that the current model of the plant clock lacks adequate activators to be rationally defined. How such activators fit into the clock system of course requires their discovery and subsequent integration to the models. As these putative activators are found, probably by molecular-genetic methods, the elements that define the core of the clock could be moved toward being considered as centrally defined. Another important consideration is to define the details of feedback signals from metabolic rhythms (Stitt and Zeeman, 2012) and their role in the redox circadian oscillator (Edgar et al., 2012). The whole clock community awaits those integrative results (van Ooijen and Millar, 2012).

## MODEL NEEDS AND PROPOSED FURTHER USES

There are several areas where modeling has yet to place the clock in a signal context of observed findings. This seems relevant as numerous transcription factors fine-tune clock parameters, implying massive signal interconnections of divergent and disparate signaling systems to and from the clock (Hanano et al., 2008). One example is that multiple phytohormones have distinct effects on clock parameters (Hanano et al., 2006; Yin et al., 2007), but to date, modeling has not explained how this feedback is plausible. Here current efforts to reciprocally link the stress hormone abscisic acid (ABA) to the clock seem particularly relevant (Legnaioli et al., 2009). This could relate physiological connections to drought and salinity on the physiology of clock performance. Additionally, as auxin signaling rises with increasing warmth (Gray et al., 1998), and as auxin application phenocopies the effect of warmth to create more stochastic noise in the oscillator (Hanano et al., 2006), this thermal-dampening mechanism could relate to an ability of increasing temperatures to increase auxin signaling flux as a modulator of circadian amplitude. Modeling this hypothesis could direct the plausibility of this. Other hormones have distinct effects on phase and period, and these could act on light signaling to the clock, but that is not yet described in mathematical terms. Modeling signaling cross-talk to and from the clock seems ripe for future investigation.

Light has two main modes to set the clock. Light intensity increases lead to periodicity decreases (Somers et al., 1998a). This speeding up of the clock by increased light perception leads to an eventual phase shift of the clock back to a correct resonance, and this is called parametric entrainment. In contrast, the discontinuous nearly immediate setting of the clock happens at dawn and needs extended light far beyond that which activates light-regulated gene expression (Millar and Kay, 1996), and this sudden clock setting is called non-parametric entrainment. In some manner, the phytochromes and cryptochromes have a role in these setting mechanisms (Somers et al., 1998a; Devlin and Kay, 2000). Interestingly, a photoreceptor complex (Mas et al., 2000) is genetically interactive in clock function (Devlin and Kay, 2000). A mechanistic hypothesis for photoreceptor input to the clock has not yet been generated. Although, it has been shown biochemically that light controls degradation of PRR7, PRR9, TOC1, and GI proteins (Mas et al., 2003; David et al., 2006; Farre and Kay, 2007; Ito et al., 2007a). These photic effects then act on outputs within a diurnal context that changes in duration throughout the season (Davis, 2002; Salazar et al., 2009; Troein et al., 2009; Guerriero et al., 2012; Song et al., 2012).

Low-fluence rate UV-B light has been shown to control development, promote photomorphogenesis, and drive gene expression (Heijde and Ulm, 2012). UVR8 and COP1 are crucial for physiological UV-B responses and entrainment of the clock by UV-B light (Feher et al., 2011). Although under supplemented UV-B light, COP1 induces ELONGATED HYPOPCOTYL 5 (HY5) and HY5 HOMOLOGY (HYH), HY5 and HYH are not required for clock entrainment by UV-B (Feher et al., 2011). With the identification of UVR8 as the UV-B receptor (Heijde and Ulm, 2012), this is another input signal to the oscillator that must also be mathematically defined as an input cue.

Interestingly, under far-red (FR) light the otherwise arrhythmic *elf3* and *elf4* mutants regain rhythmicity (Kolmos et al., 2011; Wenden et al., 2011). Phytochrome A (phyA) has been shown to be required for controlling clock-regulated gene expression under these conditions (Wenden et al., 2011), yet the effect of FR-light input to the clock is not well understood. As shade alters the red/FR ratio, this observation suggests a different clock entrainment under these environmental conditions. Here, genetics could profit from mathematical modeling.

Ambient temperature effects on the clock work in two discreet ways. In one sense, the oscillator resists changes in mean ambient temperature to run at about 24 h over a fairly wide range of temperatures. Modeling has been able to explain this as effects at transcript abundance of clock genes (Gould et al., 2006). In contrast, the daily oscillation of daytime warmth with evening coolness can set the oscillator (Somers et al., 1998b; Boikoglou et al., 2011). This form of entrainment is completely unresolved (McClung and Davis, 2010). Modeling efforts have not yet been conducted to predict inputs generated from temperature entrainment. Another point is that stress temperatures of cold can lead to oscillator arrest (Bieniawska et al., 2008). How stress temperature stops the clock is as yet non-explored in a systems sense (McClung and Davis, 2010).

Stress and metabolic signals enter the clock. Redox effects by photosynthesis, and alterations in sucrose and starch have been connected to normal oscillator function (Dalchau et al., 2011). Relations to ABA signaling appear to intersect here (Sanchez et al., 2011). How redox and carbon as photosynthesis-related processes enter the clock are not known. Modeling is likely to add useful hypotheses to this point. Other metabolites can act on oscillator parameters, including cyclic ADP ribose (cADPR), and this has been modeled (Dodd et al., 2007). Primary metabolites could act as energy intermediates, and trehalose-6-phosphate has been hypothesized to signal in homeostasis (Schluepmann et al., 2012). In contrast, secondary metabolites, such as glucosinolates that act on clock parameters (Kerwin et al., 2011), are more difficult to rationalize as just a metabolic effect. Numerous secondary compounds are perhaps probable as direct signaling molecules in clock fine-tuning. Placing all of these metabolic effects to the clock will likely be aided by informatics and systems approaches.

Clock genes in *A. thaliana* display extensive sequence variation manifested in quantitative variation within a population (Swarup et al., 1999; Boikoglou et al., 2011; Undurraga et al., 2012) and this is also seen in the monocot barley (Stracke et al., 2009; Faure et al., 2012). Furthermore, ploidy changes that are prevalent in plants also act on clock behavior at a physiologic (Ni et al., 2009) and genomic scale (Lou et al., 2012). Future clock models should be able to predict how subtle allelic variants lead to expressed-trait effects on clock parameters. As the analysis of clock-gene expression in barley did not exactly follow that of *A. thaliana* (Campoli et al., 2012), models will need to be generated that consider the evolutionary divergence between monocots and dicots. Finally, modeling is also likely to be useful in predicting how the assembly of a larger nucleus in new polyploids, and the effect of larger gene dosage, is buffered.

Moving beyond transcription, numerous clock proteins are subjected to post-transcriptional and post-translational regulation (Staiger and Koster, 2011). Phosphorylation and regulated protein degradation can be a directing force for the input of environmental signals to the oscillator (Herrero and Davis, 2012). From there, another layer within the system can be seen in regulated protein-complex assembly and the action of alteration of protein localization and DNA association capacity (Schoning and Staiger, 2005; Herrero and Davis, 2012). Together, these dynamics at the protein level need to be considered in new modeling efforts. Perhaps such an approach could lead to an improved placement of GI into the clock.

## CONCLUSION

Mathematical models of the *A. thaliana* circadian oscillator have motivated hypothesis-driven experimental studies that have largely resolved this system. In this way, the plant circadian network serves as an example for how other plant-signaling systems can profit from interactive modeling-experimental efforts.

## Conflict of Interest Statement

The authors declare that the research was conducted in the absence of any commercial or financial relationships that could be construed as a potential conflict of interest.
